# Using Silver Nano-Particle Ink in Electrode Fabrication of High Frequency Copolymer Ultrasonic Transducers: Modeling and Experimental Investigation

**DOI:** 10.3390/s150409210

**Published:** 2015-04-20

**Authors:** Adit Decharat, Sanat Wagle, Svein Jacobsen, Frank Melandsø

**Affiliations:** Department of Physics and Technology, UiT The Arctic University of Norway, Tromsø N-9037, Norway; E-Mails: sanat.wagle@uit.no (S.W.); svein.jacobsen@uit.no (S.J.)

**Keywords:** silver nano-particle ink, P(VDF-TrFE), high frequency copolymer ultrasonic transducer, transducer printing material

## Abstract

High frequency polymer-based ultrasonic transducers are produced with electrodes thicknesses typical for printed electrodes obtained from silver (Ag) nano-particle inks. An analytical three-port network is used to study the acoustic effects imposed by a thick electrode in a typical layered transducer configuration. Results from the network model are compared to experimental findings for the implemented transducer configuration, to obtain a better understanding of acoustical effects caused by the additional printed mass loading. The proposed investigation might be supportive of identification of suitable electrode-depositing methods. It is also believed to be useful as a feasibility study for printed Ag-based electrodes in high frequency transducers, which may reduce both the cost and production complexity of these devices.

## 1. Introduction

The polymer vinylidene fluoride (PVDF) and the copolymer obtained from vinylidene fluoride and trifluoroethylene [P(VDF-TrFE)] have been used extensively as materials for piezo- and pyro-electrical sensors and in piezoelectric transducers [1,2,3]. The copolymer is often preferred since it can be deposited directly onto a substrate by various methods (e.g., spin coating, bar coating, dip coating and spraying). It was recently shown that [P(VDF-TrFE)] also can be screen-printed into all-printed devices such as touch sensors [4]. In general, printed sensors and the merging of printed devices and electronics, can have a large potential for cost reduction in production [5,6], but a number of challenges have to be solved to efficiently integrate what is typically a large number of different materials (both organic and inorganic).

For many sensor and transducer applications, the properties of conductive layers or electrodes are important, e.g., in terms of conductivity and transparency. In high frequency ultrasonic transducers, for example, electrodes have typically been produced by plasma and/or vacuum methods like sputtering or vacuum deposition. These methods are difficult to integrate efficiently in printing processes, and thereby limit the ability for mass production. It is therefore often preferable to use printable conductive inks (polymer- or metal-based) as electrode materials. Printable conductive polymer electrodes have previously been applied in high frequency (HF) ultrasonic transducers yielding very good impedance matched to piezoelectric films [7]. However, their low electrical conductivity affects the transducer sensitivity [8]. For electrodes used in the active part of an ultrasonic transducer, the electrode thickness also plays an important role in addition to the conductivity [9]. This is due to the additional mass introduced by the electrode material, which typically can be significantly higher when using a conductive metal-based ink as a replacement for sputtering or vacuum deposition.

In the current manuscript, we have investigated both theoretically and by experiments the effects induced by a typical printed electrode in a high frequency ultrasonic transducer. A silver (Ag)-based nano-particle ink was used as the test electrode material, and different dried thicknesses of this material were applied by spin coating and drying a various numbers of layers to obtain thicknesses typical of both ink-jet and screen printing. Nano-particle inks like the one under test have previously shown promising characteristics for flexible device-electrode fabrication (e.g., good conductivity, adhesive strength and large-scale production possibilities) [10,11,12,13,14]. However, for electrodes on P(VDF-TrFE) the temperature has to be maintained below 140 °C during the sintering process, which to some extent, limits the conductivity of the material [15,16]. Our focus have therefore been to obtain electrodes with sufficient conductivity but at the same time, not to impose severe electrode mass loading, effects from e.g., times of internal reflections and bandwidth reduction. The effects on the transducer properties imposed by a backing layer were also investigated.

## 2. Basic Model Theory

**Figure 1:**
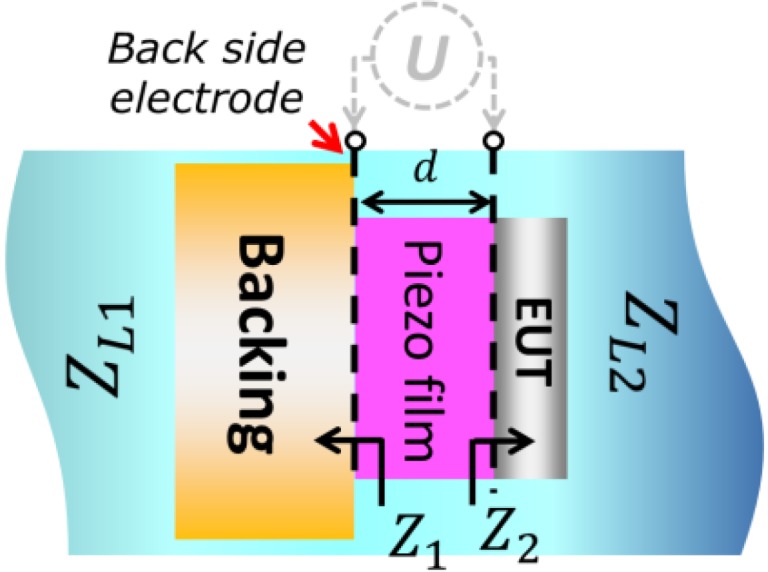
Schematic drawing of the transducer containing a piezoelectric film and acoustic slabs.

An ultrasonic sensing system is basically composed of multilayer elements including piezoelectric film, electrodes, backing and a load medium. In the current study, we have focused on the mass loading imposed by having a thick layered electrode on the front side of the transducer. This electrode under test (hereafter denoted as EUT) is shown in Figure 1 together with a thin back side electrode (assumed to impose no mass loading), a finite backing material, and infinite loading materials on the back and front side. Many models used for analyzing transducer properties have previously been reported [17,18,19]. The one which will be used here is the impedance matrix model of a three-port network.

**Figure 2:**
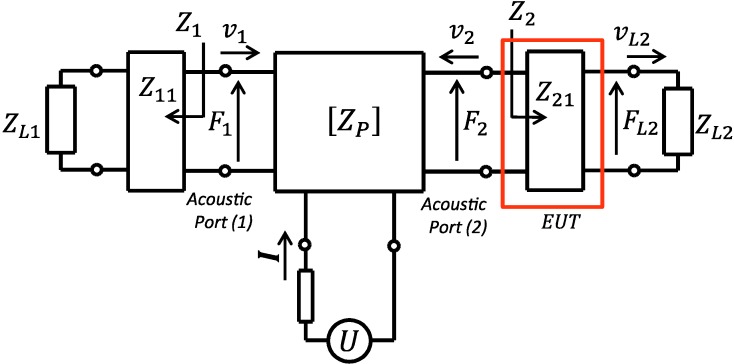
Schematic drawing of a three port network transducer model. The model includes a piezoelectric film in cascade with one electric port and two acoustic ports terminated by acoustic loads$\;Z_{L1}$ and$\;Z_{L2}$.

The system configuration in Figure 1 can essentially be modeled as a three-port network as shown in Figure 2. In the model, the EUT layer was separated from the piezoelectric element and the layer assumed as one of the delay lines which imposes a mass loading on the thin front side electrode. From the figure, the piezoelectric film has the force $F_{1},$ $F_{2}\;$and incoming particle velocity$\;v_{1}$ and $v_{2}$ at the acoustic port. Each acoustic port is connected to the external matter of equivalent acoustic impedance $Z_{1\;}$and $Z_{2}$ for port 1 (back side) and port 2 (front side), respectively. We apply electrical voltage (*U*) and current (*I*) on the electric port.

### Model of Acoustic Delay Line

To estimate the effect caused by the acoustic layer, the slabs of acoustic impedance$\;\;Z_{11}$ and $Z_{21}$ (at the acoustic terminal ports, see Figure 2) were modeled as a delay line with phase constants in the materials as $\varphi_{11}$ and $\varphi_{21}$, respectively. Determination of $Z_{1\;}$or $Z_{2}$ is obtained by impedance transformation of these acoustic layers cascading with the terminated load which can be calculated from the well-known impedance transformation [19]. For instance, in case the acoustic layer at port 2 (EUT) is terminated by air $Z_{L2}=Z_{air}\approx 0$, the equivalent acoustic impedance looking from port 2 of the transducer is:

(1) $$Z_{2}=Z_{21}\frac{Z_{L2}\cos\varphi_{21}+iZ_{21}\sin\varphi_{21}\;}{Z_{21}\cos\varphi_{21}+iZ_{L2}\sin\varphi_{21}\;}=iZ_{21}\tan\varphi_{21}$$

for which $\varphi_{21}\;$=$\;\;k_{21}L_{21}$, where$\;\;k_{21}\;$is the wavenumber in the front delay line (EUT) and $\;L_{21}\;$is the thickness of the EUT layer, $Z_{21}\;$is the acoustic impedance of EUT and $Z_{L2}\;$is the terminated load impedance.

## 3. Constituent Layer Dependence of Transmitted Power Frequency Response

The transducer emits power as a function of frequency which essentially is influenced by its constituent material properties. In this section we derive a theoretical model of such an effect and compare it to COMSOL numerical modeling. To analyze the transmitted acoustic power, we start by calculating the particle velocity (v) generated by the piezoelectric film.

### 3.1. Particle Velocity

The relation of v and equivalent acoustic impedances at the transducer port can be derived from the matrix of three-port network as [20], for ${𝒵}_{n}=AZ_{n}\;\left(n=1,\;2\;\text{and}\;P\right)$:

(2) $$v=\frac{hC_{o}\left(i{𝒵}_{1}-{𝒵}_{P}\text{tan}\left(\frac{\varphi_{P}}{2}\right)\right)U}{\left({𝒵}_{1}+{𝒵}_{2}\right){𝒵}_{P}\left(\cot\varphi_{P}-\frac{K^{2}}{\varphi_{P}}\right)+i\left({𝒵}_{1}{𝒵}_{2}+{𝒵}_{P}^{2}-\frac{2K^{2}}{\varphi_{P}}{𝒵}_{P}^{2}\text{tan}\left(\frac{\varphi_{P}}{2}\right)\right)}$$

where $\varphi_{P}$ = kd, where k is a wavenumber in the piezoelectric film, d  is film thickness, K  is electromechanical coupling coefficient, A is the effective area of an acoustic port (assumed equal for both ports), $Z_{P}\;$ is the acoustic impedance of the piezoelectric film, $C_{o}$ is the clamped capacitor of the piezoelectric film and h is the piezoelectric coefficient (here it is $h_{33}$).

### 3.2. Acoustic Power Transmitted to Load

The mean power emitted at the load port, which has equivalent acoustic impedance of Z, can be expressed as:

(3) $$〈P〉=\frac{1}{2}AZ{\left|v\right|}^{2}$$

where A is effective area of load port and v is particle velocity generated in the piezoelectric film (see Equation (2)). Equations (2) and (3) show that the emitted power at the load layer is influenced by the impedance of both backing and load port.

The effect caused by an EUT layer is evaluated by calculating the power transmitted through the layer towards the load. When the front electrode (EUT) thickness is significant, as in Figure 1, acoustic power delivered to the load (here is $Z_{L2}$) can also be calculated by Equation (3). Alternately, we use $Z_{L2}$ and $v_{L2}$ as the impedance and particle velocity, respectively, where $v_{L2}$ is the particle velocity at EUT layer and $Z_{L2}$ interface. The EUT delay line with impedance $Z_{21}$ can be seen as a mechanical two-port network with $v_{2}$ and $v_{L2}$ as particle velocities at the left and right port, respectively (see right network arm of Figure 2). The value of $v_{L2}$ can be obtained from the EUT two-port network matrix [21]. The EUT slab thickness, however, is considered to be relatively thin compared to the wavelength in material which gives $\;\varphi_{21}\ll 1\;$and results in the term$\text{ tan}\varphi_{21}\approx \varphi_{21}\approx k_{21}L_{21}$. Thus, the EUT delay line transmission matrix $T_{21}$ can be simplified to:

(4) $$T_{21}=\;\left[\begin{array}{cc} a_{t21} & b_{t21}\\ c_{t21} & d_{t21} \end{array}\right]=\;\left[\begin{array}{cc} 1 & iAZ_{\begin{array}{c} 21 \end{array}}k_{21}L_{21}\\ \frac{ik_{21}L_{21}}{AZ_{21}} & 1 \end{array}\right]$$

From the EUT two-port network, we obtain:

(5) $$\left[\begin{array}{c} F_{2}\\ -v_{2} \end{array}\right]=\;\left[\begin{array}{cc} a_{t21} & b_{t21}\\ c_{t21} & d_{t21} \end{array}\right]\left[\begin{array}{c} F_{L2}\\ v_{L2} \end{array}\right]$$

and since $Z_{L2}=\frac{F_{L2}}{v_{L2}}$.

Then the particle velocity in $Z_{L2}$, is:

(6) $$v_{L2}=\frac{-v_{2}}{\left(c_{t21}Z_{L2}+d_{t21}\right)}$$

## 4. Constituent Layer Dependence of Transducer Electrical Properties

In this section we present some analytical expressions necessary for understanding the influence of the acoustic layer on the transducer electric properties. Analytical models were also derived based on the previously mentioned three-port network. Some of these models will be compared to the experimental results later in the paper.

### 4.1. Transducer Electrical Impedance Model

The electric impedance (or inverse admittance) of loaded transducers can be determined from the impedance matrix which analytically can be expressed using Equation (1.56) from [20]. Using trigonometry identities and given that $\;r_{u}=\;Z_{1}Z_{2}/Z_{P}^{2}$, $\;r_{v}=i\left(\;Z_{1}+Z_{2}\right)/2\;Z_{P}$ and $\theta_{P}=\varphi_{P}/2$ , Equation (1.56) in [20] can be modified into:

(7) $$Z_{e}=\frac{1}{Y}=\frac{1}{i\omega C_{o}}\left[1-\frac{K^{2}/\theta_{P}\;}{\cot\theta_{P}-\frac{\;(\;r_{u}+\;r_{v}\text{ tan}\theta_{P})}{\left(\;r_{v}-\text{ tan}\theta_{P}\right)}\;}\right]$$

in which $C_{o}=\frac{\varepsilon A}{d}$, where ε is the dielectric permittivity.

### 4.2. Effect of EUT Variation on Electrical Properties

**Figure 3:**
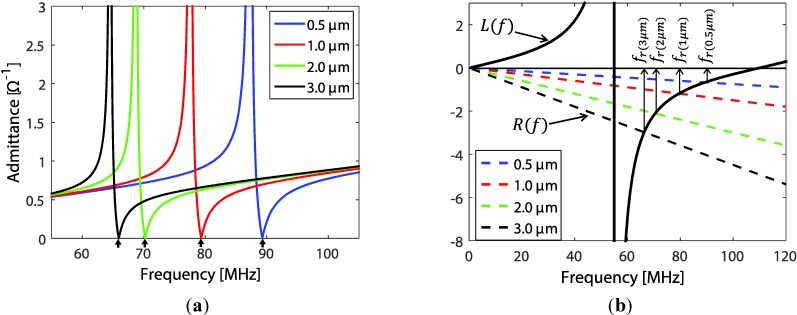
The shift in resonance frequency for transducers with different EUT thicknesses. (**a**) Transducer admittance with varying EUT thickness $L_{21}$ and (**b**) graphical illustration of the trajectory of resonant frequency when varying EUT thickness.

As a simplified illustration, the admittance of stand-alone transducer film (no backing material and $Z_{L2}$) with varying EUT thickness (which is the $L_{21}\;$ layer) is illustrated through resonant frequency shifting. For this case we have $\;Z_{1}=0$ (since $\;L_{11}=0,\;$ also neglecting the rear electrode thickness) and$\;Z_{2}$ is as in Equation (1). Assuming no loss in the piezoelectric film, using the expression of $\;Z_{1}$ and $\;Z_{2}$ for Equation (7) together with the material constants in Table 1, the plot of the admittances is shown in Figure 3a. A shift in resonance frequency $\left(\;f_{r}\right)$ (indicated by lower arrows) is observed and represents the behavior of the transducer admittance response to $L_{21}$ variations. Alternately, with the resonant condition, $\;Z_{e}\Rightarrow \infty \;$ and Y⇒0, the denominator of the rear term in the parenthesis of Equation (7) equals zero. For $\;\varphi_{21}\ll 1\;$ and using trigonometry identities, the denominator term can be rewritten as:

(8) $$\tan\left(\frac{2\pi \;f_{r}}{V_{P}}d\right)=-\frac{2\pi \;f_{r}}{V_{P}}∙\frac{\rho_{21}L_{21}}{\rho_{P}}$$

where $V_{P}$ and $\rho_{P}$ are the phase velocity and mass density of the piezoelectric material, respectively and $\rho_{21}$ is the mass density of the EUT material (e.g., silver). The intersect plot of left (*L*) and right term (*R*) graphs of Equation (8) is shown in Figure 3b as a function of frequency.

**Table 1:** Material constants and parameters.

Parameter	Value	Parameter	Value
$\varepsilon_{r}$	6.2	** K	3.9 (PEI), 4.8 (25 μm), (50 μm)
$tan\delta_{m\left(P\right)}$	0.2 (PEI),(PI:25 μm), 0.15 (PI:50 μm)	α	0.088 (PEI), 0.11 (25 μm), 0.105 (50 μm)
U [V]	5	$tan\delta_{m\left(PI\right)}$	0.05 (25 μm), 0.01 (50 μm)
$c_{33}\left[N/m^{2}\right]$	$1.11\times 10^{10}$	$V_{PI}\;\left[m/s\right]$	2000 (25 μm), 2150 (50 μm)
$\rho_{P}\;\left[Kg/m^{2}\right]$	1680	$\rho_{PI}\;\left[Kg/m^{2}\right]$	1430
d [μm]	12	$tan\delta_{m\left(PEI\right)}$	0.003
D [mm]	2.5	$\rho_{PEI\;}\left[Kg/m^{2}\right]$	1270
K	0.2247	$V_{PEI}\left[m/s\right]$	2180
$h_{33}\left[m/V\right]$	$-0.162\times 10^{-10}$	$L_{11}\;\left[\mu \text{m}\right]$	25 and 50 (PI), 850 (PEI)

The intersecting trajectory of these two lines presents graphically the shifting of ${\text{ f}}_{\text{r}}$, which also roughly illustrates the saturation area and boundary limit of the ${\text{ f}}_{\text{r}}$ shifts.

### 4.3. Effect on Electrical Property of Lossy Transducer with Constituent Layer System

Copolymer piezoelectric materials possess two significant loss properties which may be modeled by the dielectric loss factor $\;\tan\left(\delta_{e}\right)$ and mechanical loss factor $\tan\left(\delta_{m}\right)$ [22]. Accounting these loss factors into transducer response modeling was initially proposed by [23]. Additionally, in case the dielectric permittivity of the clamp capacitance $C_{o}$ is assumed frequency dependent. To implement the effect, we used a constant phase (CP) model [24], $\varepsilon \left(\omega \right)=K{\left(j\omega \right)}^{-\alpha }$ where K and α are two model parameters, for which the parameter value selection have been described in previous work [8]. In lossy material, the acoustic impedance companying a mechanical loss can be determined from Z= ρV  which for piezoelectric material is given by:

(9) $$V=V_{P}=\sqrt{\frac{c^{D}}{\rho_{P}}}\;$$

for which:

$$c^{D}=c^{E}\left(1+\frac{K^{2}}{1-K^{2}}\right)$$

$$c^{E}=c_{33}\left(1+j\tan\left(\delta_{m\left(P\right)}\right)\;\right)$$

where $c^{D},\;c^{E}$ are stiffness coefficients at constant electric displacement and electric field, respectively, $c_{33}$ is the elasticity in the direction of thickness axis and $\tan\left(\delta_{m\left(P\right)}\right)$ is the mechanical loss factor of the copolymer.

In most applications, polymer transducers are fabricated with non-piezoelectric layers (e.g., backing substrate or front layer) in order to support a piezoelectric-film structure or to damp out the signal tail (e.g., in short pulse applications). These non-piezoelectric layers will also introduce an additional mechanical loss factor. Thus, to improve model reliability, we have taken such effects into account in the transducer model for example in backing polymer, material phase velocity companying a mechanical loss $V_{backing}^{\prime }=V_{backing}{\left(1+j\tan\left(\delta_{m\left(backing\right)}\right)\;\right)}^{1/2}$ where $V_{backing}$ is backing material phase velocity excluding loss and $\tan\left(\delta_{m\left(backing\right)}\right)$ is mechanical loss factor of the backing.

For transducers with backing substrate, the equivalent acoustic impedance at the backing port $\;(Z_{1})$ is also defined by Equation (1) and given by $\;Z_{1}=iZ_{11}\tan\varphi_{11}\;$(for$\;Z_{L1}$ = air). For the EUT layer, the acoustic impedance will be $Z_{2}=iZ_{21}\varphi_{21}$ (for $\;\varphi_{21}\ll 1$ and $\;Z_{L2}$ = air).

## 5. Prototyping

Two polymer substrates used as a non-piezoelectric material were polyethylenimine (PEI) and polyimide (PI). These two polymers are available as commercial products of various types such as PEI sheets and PI in rolls. The piezoelectric-sensitive film was made from PVDF copolymer which was P(VDF-TrFE) powder (77:23 in molar ratio).

Preparation of P(VDF-TrFE) solution for piezoelectric film development was done by blending 3.5 mL of DMF (dimethylformamide) solvent with 1 g of 77:23 molar ratio P(VDF-TrFE) copolymer powder. This solution was then mixed using an ultrasonic disperser to completely dissolve the powder into a viscous fluid. Fabrication was initiated by preparing a PEI substrate with size 50 × 50 mm^2^ (also acting as a backing material). The polymer backing substrate was treated with plasma using a low pressure air atmosphere. This was done to promote good adhesion at the interface surface prior to rear electrode implementation (for mass-production purposes, the plasma treatment can be replaced by chemical treatment [25]). The first (rear) electrode layer was made by sputtering the silver (Cressington 208HR) on the pre-treated polymer substrate. The Ag layer with nominal thickness of 50 nm was then patterned by photolithography (using pattern printing on transparent paper as a mask) and wet-etching to obtain the desired electrode diameter of 3 mm. The layer of P(VDF-TrFE) solution was spin coated over the patterned electrode to achieve a film thickness close to 12 µm; two different spinning velocities (1000 rpm and 3000 rpm) were used for 9 s and 6 s, respectively. Also, to obtain a similar film thickness for four transducer elements, the substrate was centered (at the center point of the four elements) before spinning. After spinning, the substrate was degassed in a 1 mbar vacuum atmosphere to vaporize the solvent. The film thickness was estimated using a KLA/Tencor P6 surface profiler as explained in [7].

**Figure 4:**
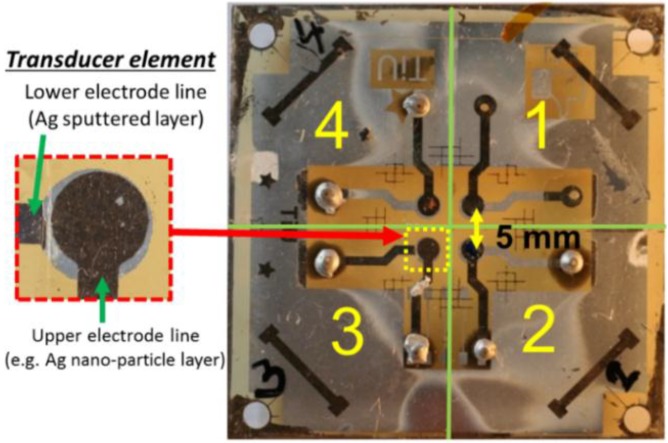
Image of the transducer panel containing four different EUT thickness transducer elements and area divisions for tape covering. The inset image shows an enlarged view of the transducer element.

In order to crystalize the piezoelectric film, the sample was annealed at a temperature of 130 °C for 6 h. After annealing, the P(VDF-TrFe) surface was treated with plasma. The last transducer layer is a front electrode (EUT) made from Ag nano-particle ink (ALDRICH/719048) provided by Sigma Aldrich (St. Louis, MO, USA). The layer was implemented by spinning the ink on the piezoelectric film, which afterwards was sintered at the temperature of 130 °C for 1 h to enable the conductivity of the layer. To create a thicker EUT layer, the layer was repeatedly spun with the ink. To be able to make several different thicknesses on the same substrate, some areas were covered with tape which allows selective-thickness deposition of the ink on the substrate. For instance, to produce several different thickness layers, we first deposit ink covering the whole upper-side of the copolymer layer after annealing, then for the second ink deposition layer, area 1 was taped allowing ink to cover only areas 2, 3 and 4 (see Figure 4). Using the same approach, one can create the remaining layers. After that the layer was patterned to achieve an electrode diameter of 2.5 mm (by a similar process as described for patterning the rear electrode). Figure 4 also shows the transducer substrate which contains four transducer elements with an element pitch of 5 mm. In between the processing steps, the Ag nano-particle ink thickness was also measured using our KLA/Tencor P6 surface profiler. This instrument was also used to measure the rms surface roughness, typically ranging from 0.09 to 0.24 µm on the top of the transducer aperture.

For a thin substrate such as PI, the thermal release adhesive tape (Nitto Denko, Inc., Osaka, Japan, courtesy of Teltec GmbH, Mainhardt, Germany) was used to support the thin film structure during the layer developing processes. The electrical connections out of the transducer were made by joining pin connectors to the electrode layer with conductive epoxy.

To make P(VDF-TrFE) films piezoelectric, the elements were poled using a high voltage AC source at room temperature (10 periods with amplitude 825 V and frequency 0.25 Hz). The proposed AC poling schedule involving multiple dipole switching, which is often preferred compared to a DC poling, e.g., due to an enhanced transducer response and improved homogeneity of the poled area [7,8,26].

## 6. Characterization

An electrical LCR analyzer (E4982A, Agilent, Santa Clara, CA, USA) with a 1–300 MHz frequency range was used to characterize the transducer electrical properties. To measure the transducer parameters, the instrument generated equally coherent stepped frequency wave magnitudes over the programmed frequency band consequently collecting the detected response signal from the transducer for calculation processing. To alleviate the capacitive effect introduced by wire and transducer conductive line, transducer-like calibration kits were made. The calibration kits were built to compensate for the pin through the legs to the piezo-sensitive area. The thickness of the patterned EUT element was measured using a KLA/Tencor P6 surface profiler.

**Figure 5:**
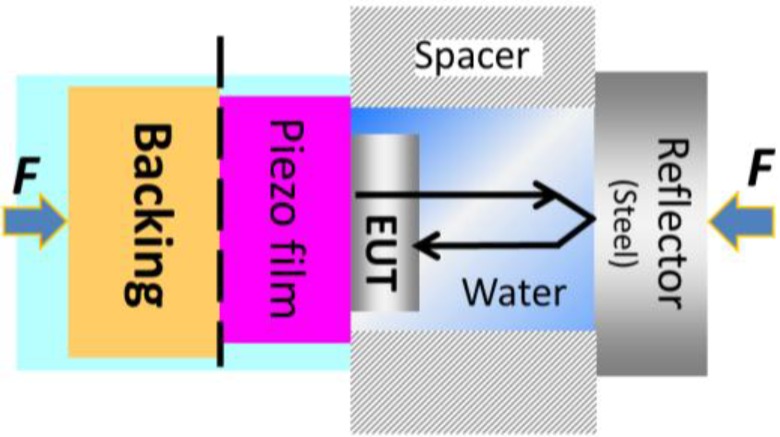
Illustration of the acoustic response signal characterization of the wave travelling through the EUT layer.

Transducer functionality was tested by measuring the acoustic response signal. Unlike the LCR analyzer system, the measurement setup used a pulse-echo system excited by signal of Gaussian 2nd derivative as described in [8] with a pulse width σ=2.94 ns. The acoustic response signal of the transducers was measured. To measure the wave passing through the EUT layer, we consider only the reflected wave from the front side instead (which distinguished by calculating the time delay) as depicted in Figure 5.

From the figure, the spacer is a microscope glass of 1 mm thickness and the reflector is highly polished surface steel of 1.5 cm thickness which ideally yields a total reflection of the wave. For a coupling media of acoustic wave, the front gap was filled up with distilled water and the setup was stabilized by applying clamping force on both side of the setup. Here, one should notice that the acoustic wave will travel through the EUT layer twice before being detected by piezoelectric film.

## 7. Results and Discussion

### 7.1. Modeling Results of the Effect on Transmitted Power Frequency Response

**Figure 6:**
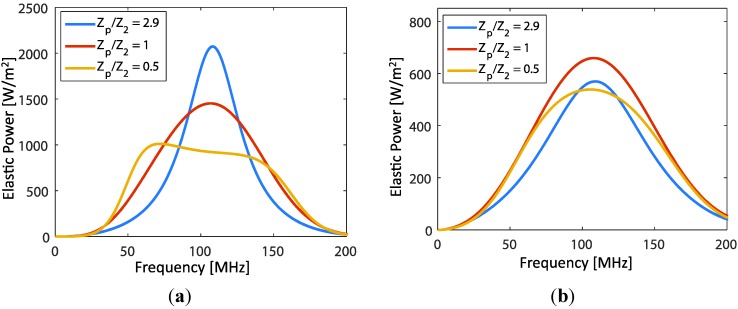
The frequency response of transmitted acoustic power at port 2. (**a**) $Z_{1\;}=0$ (no backing material, e.g., air backing) and (**b**) with $Z_{1\;}=$ polymer substrate impedance (e.g., PEI polymer backing).

In this section we present results of the study in the Section 3. Initially, two different configurations of the transducer (no backing material and with polymer backing) were studied. In the model, we assume that EUT layer thickness was negligible. We used the material constants and loss factors as shown in Table 1. With front load variations (in this case $\;Z_{2}=Z_{L2}$), the transmitted acoustic power (TAPF) per unit area frequency response was determined by dividing Equation (3) by A. Figure 6a shows the influence of the front load variation on the TAPF of the transducer with no backing material (therefore $Z_{1}=\;$air). The variations are expressed in term of $Z_{P}/Z_{2}$. The transducer produces the same trend as in Section 1.4.1 of [20] (higher impedance ratios yield higher magnitude, but narrower bandwidth). However, in the polymer backing case (Figure 6b), the response characteristics are different. The peak magnitude is accompanied by a broad bandwidth when $Z_{P}/Z_{2}=1$ (matched impedance). The widest bandwidth occurs when $Z_{P}/Z_{2}=0.5\;$. In this case, marginal difference of the magnitude, also the bandwidth, among the impedance ratios of 0.5, 2.9 and 1 are observed. Comparing the results of two transducers, the TAPF characteristics vary significantly. For instance, when loaded by water $\left(Z_{P}/Z_{2}=2.9\right)$, the transmitted power magnitude and the bandwidth of air backing and polymer backing transducer are $2073\;W/m^{2}$ with 44 MHz, and $570W/m^{2}$ with 81 MHz, respectively. Thus, to obtain a large signal bandwidth, the backing substrate is important, but at the cost of decreasing the transducer sensitivity.

**Figure 7:**
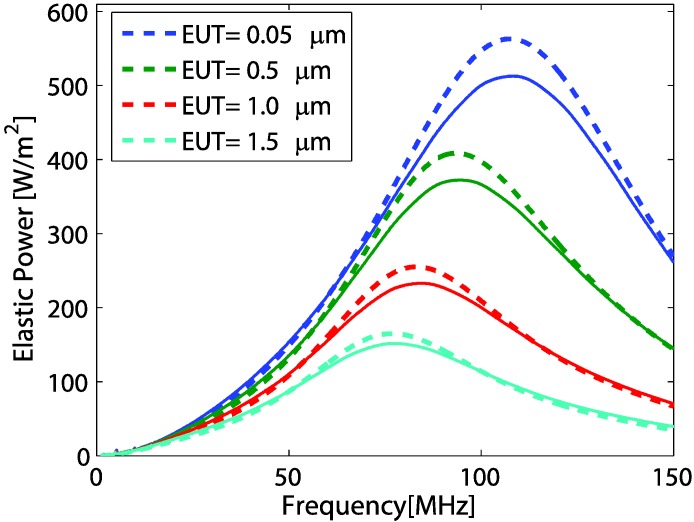
Comparison of transmitted power frequency response obtained from analytical model (dashed line) and COMSOL model (solid line) for different EUT thickness.

For a significant EUT thickness, the effects due to electrode thickness variations were studied. As in Figure 2, we assume that the polymer backing transducer was operated with a front load $\;(Z_{L2})$ of water. In this case, the polymer backing length was infinite and the rear electrode thickness was neglected. Thus, $\;\;Z_{1}=Z_{11}$ is the impedance of the backing polymer. With the condition $\;\varphi_{21}\ll 1\;$and $Z_{21}$ is much larger than $\;Z_{L2}$, the impedance transformation at port 2 can be simplified to $Z_{2}=Z_{L2}+iZ_{21}\varphi_{21}$. A variable $v_{L2}\;$used in Equation (3) was defined by Equation (6). Thus, with the EUT thickness variation, using Equation (3) and $Z_{L2}$ of water impedance, the TAPF at the load $\;Z_{L2}$ can be plotted as shown in Figure 7.

In the figure, results from numerical modeling by COMSOL simulation software are also plotted. Variations of TAPF characteristic, influenced by thickness variation, are depicted in Table 2. Here, we notice that the magnitude of acoustic power, the peak frequency and the 3dB bandwidth decreased with increases in the EUT thickness. Thicker EUT layers act as energy band pass filters with more effects at higher frequencies (right part). The thicker layer provides a higher conductivity, but will cause the reduction of bandwidth with down-shift in frequency peak magnitude. Similar effects, but resulting from the sputtered electrode transducers were also experimentally observed in [9].

**Table 2:** Characteristics of transmitted power frequency response obtained from analytical model.

EUT Thickness (µm)	Peak		3 dB-BW (MHz)
	Frequency (MHz)	Power Mag. W/m^2^	
0.05	107.4	563	80
0.5	93.4	409	74
1	83	255	68
1.5	76.8	165	64.3

### 7.2. Effects on Electrical Properties

Direct measurement of the effect on the transducer properties in acoustic domain is relatively complicated (e.g., measurement of particle velocity in the material). It is more convenient to demonstrate the effect by comparison of analytical model and the experiment via the electrical domain.

#### 7.2.1. Analytical Results

**Figure 8:**
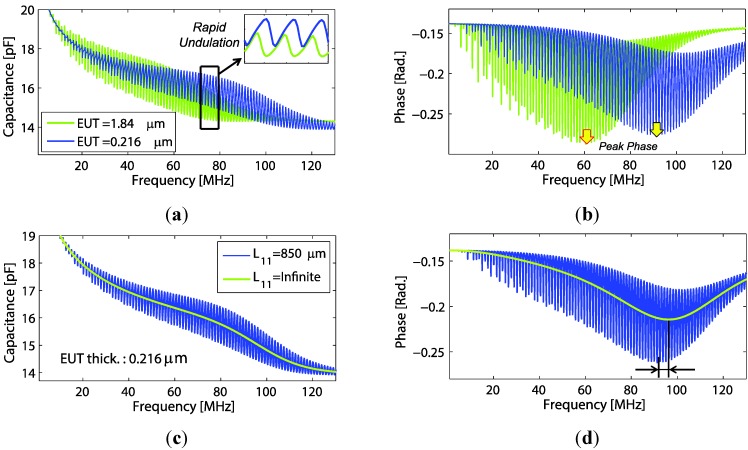
Comparison of electrical properties for transducers with PEI backing. The figures show (**a**) capacitance and (**b**) phase for different EUT thickness (with constant backing thickness = 0.85 mm); and (**c**) capacitance and (**d**) phase for different backing layer thickness $\;(L_{11\;})$.

Computed results of electrical properties based on the theory in Section 4 are presented. The transducer fabricated on PEI polymer substrate thickness of 850 μm was used in this calculation. Acoustic impedances $\;Z_{1}$ and $\;Z_{2}\;$and also the material loss factor were defined as in Section 4.3. Using Equation (7), the capacitance and phase of the polymer backing transducer with EUT thickness variations can be plotted as shown in Figure 8a,b. From the figure, rapid undulations in its envelope pattern are observed. What causes of the undulation and its characteristics were discussed in previous work [8]. In Figure 8, a rising tail at the low frequency part of the capacitance occurred when a constant phase model was included in the calculation. Here, one should notice that pattern shift of the capacitance undulation envelope is in accordance with the peak-phase frequency shift (Figure 8b), which are both proportional to the EUT thickness variation. It is also of interest to compare the effects when backing length is infinite $\left(L_{11\;}\to \infty \right)$. For this condition, $Z_{1\;}$becomes purely resistive and equal to the characteristic impedance of the backing material. Thus, by using Equation (7), plot of capacitance and phase of the transducer with finite and infinite backing slabs are compared (Figure 8c,d) The resulting curves of infinite backing length are smooth and have the same trend as the finite ones, but marginally different peak-phase frequencies are observed (90 MHz and 96 MHz).

#### 7.2.2. Experimental Results

**Figure 9:**
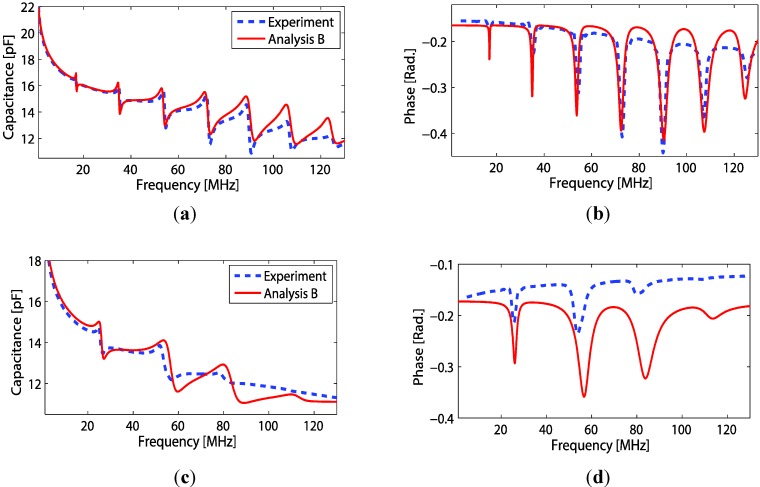
Comparison of analytical and experimental data for a transducer using a PI-backing substrate with a back side sputtered electrode. The figures show (**a**) capacitance and (**b**) phase with a Ag-sputtered front electrode and a 50 μm PI backing. Figures (c) and (**d**) show corresponding results using a 25 μm PI-backing substrate and a Ag-nano particle ink front electrode.

Several configurations of the polymer backing transducer were implemented and thereafter electrically poled before measuring the electrical properties. Most transducers were fabricated on PEI polymer thickness of 850 μm. As seen from the modeling, the substrate layer also influences the transducer properties. Thus, a couple of transducers were fabricated on the thin PI substrate (thickness of 25 μm and 50 μm). Figures 9a,b show the measured properties of the transducer whose both side electrodes were made by conventional metal sputtering (silver). The transducer with (both) electrode thicknesses of 50 nm was fabricated on PI polymer substrate of 50 μm thickness. Using material constants as in Table 1 for Equation (7), a comparison of the mathematical model to the experimental measurements can be made. The phase velocities of PI given in Table 1 were obtained by adjusting either Young’s modulus or Poisson’s ratio within the parameter ranges reported for the material, until a reasonable good agreement between the analytical and experimental curves have been achieved. One should notice that since the analytical model is one-dimensional, so an adjustment of any of these material values will give the same results as long as the longitudinal phase velocity is the same. For the PI polymer backing transduce thickness of 25 μm, the comparison plots are shown in Figure 9c,d. The transducer front electrode thickness of 0.98 μm was made of Ag nano-particle ink. For both transducers, better agreement between experimental and analysis data occurs in the lower frequency region. The observed differences in the high frequency regime can be due to calibration errors in the impedance measurements and/or frequency dependent errors occurring from e.g., the permittivity model or from other analytical models.

**Figure 10:**
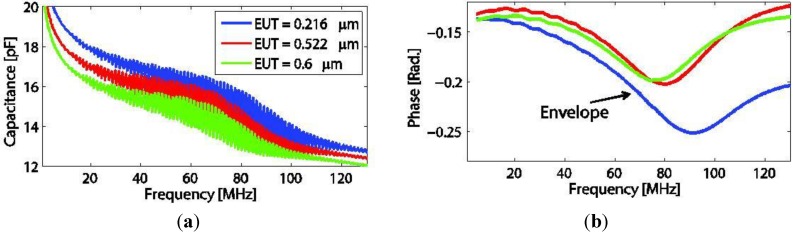
Capacitance (**a**) and phase (**b**) measurements of the three transducers with different EUT thicknesses.

Transducers with different EUT thicknesses were fabricated on PEI polymer backing with thickness of 850 μm. Nine transducer elements were successfully poled. Examples of electrical property measurements of three transducers are shown in Figure 10a,b.

A rapid undulation occurs in both capacitance and phase in a same way as seen in Section 7.2.1, but for the phase, the data were processed to only plot the envelope. From both figures, the capacitance pattern of the measurement and its responding phase peak shift is inversely proportional to the EUT thickness value.

**Figure 11:**
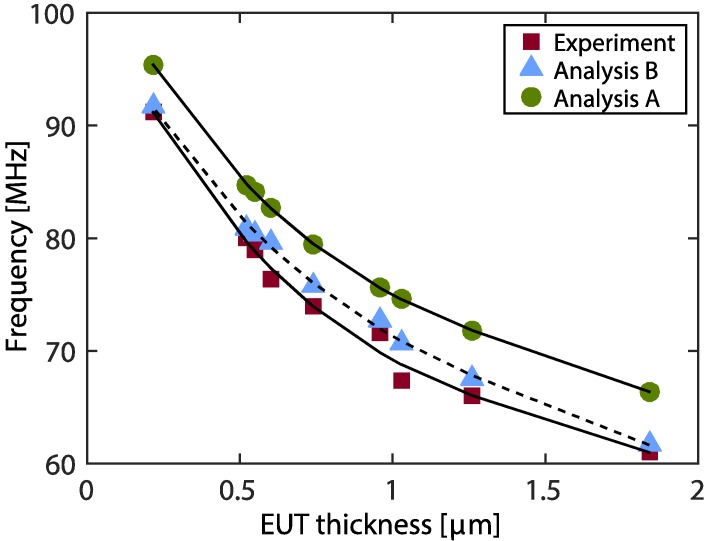
Shifting of peak phase frequency versus EUT thickness.

Plots of peak phase frequency versus EUT thickness are shown in the Figure 11. The figure shows a comparison of the analysis A determined from Equation (7) with the condition of infinite backing length $\left(L_{11\;}\to \infty \right)$, analysis B determined from Equation (7) with the actual backing length (850 μm) and the experimental data. Considering the analysis data, as we see for a thin EUT layer, the peak phase frequency changes dramatically for the variation of thin EUT layer and slows down as the EUT layer become thicker. Similar properties were observed in the case of air backing (Section 4.2). Experimental data show the same tendency as analysis B even though some values are disparate. Also, the experimental data are not smoothly sorted. A plausible explanation is that it results from variations of the thickness of piezoelectric film (*i.e.*, errors from film manufacturing) which yield different transducer resonant frequencies. All experimental data have a lower frequency value (compared to Analysis B) for each EUT thickness. Also comparing different backing thicknesses (Analysis A and B), the infinite backing transducer yields a higher phase peak frequency for each EUT thickness.

**Figure 12:**
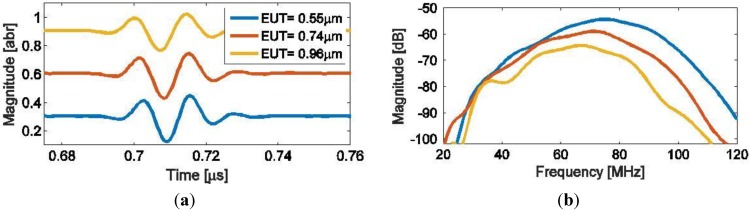
Measured acoustic responses for three transducers with different EUT thicknesses. Here the pulses are shown in time-domain (**a**) with a small DC value added for separation, and the corresponding frequency spectrum of the signals (**b**).

Finally, three transducers with different front electrode thicknesses (all with the Ag nano-particle ink) were characterized acoustically with the measurement set up as described in Figure 5. Examples of the acoustic responses obtained from a metal reflector are shown in Figure 12 both in the time (Figure 12a) and frequency domains (Figure 12b). The frequency responses (Figure 12b) show a decreasing magnitude as the EUT thickness increases. From the figure, it is easy to determine a central frequency *f_c_* where the maximum response occurs, and 6 dB bandwidth for each transducer, which are listed in Table 3. Figure 12 shows the same tendency as for the elastic power previously shown in Figure 7, *i.e.*, a decrease in central frequency *f_c_* and magnitude with an increasing EUT thickness.

**Table 3:** Measured acoustic performance of transducers with different EUT thicknesses.

EUT Thickness (µm)	Peak Frequency (MHz)	Bandwidth (6 dB) (MHz)	Bandwidth %
0.55	75	36.6	49
0.74	71.4	33.6	47
0.96	67	34.2	51

It is interesting to compare the performance of our experimental nano-silver transducers (e.g., as summarized in Table 3) with transducers having conventional sputtered electrodes. The performance of transducers with comparable film thicknesses have been reported previously, although with some differences, e.g., in terms of substrate/backing material, sizes of the effective area, and front side medium.

For example, in [27], unfocused P(VDF-TrFE) copolymer transducers with an upper of sputtered aluminum electrode and a lower sputtered chromium/gold/ chromium electrode were built on top of a similar substrate (PEI). These transducers with the same thickness (12 µm) yielded a center frequency of 72 MHz and a 6 dB bandwidth of 70%. These parameters are comparable to the one listed in Table 3.

Focused transducers made from PVDF and P(VDF-TrFE) on epoxy and aluminum backings with sputtered electrodes from chromium/gold have been studied in [2,9]. In [9], using an epoxy backing with film thickness of 10 µm, the authors obtained center frequencies varying from 35 to 44 MHz and a 6 dB bandwidth from 63% and 131% depending on differences in the measurement conditions. The work reported in [2] with a film thickness 10 ± 2 µm and aluminum backing showed a center frequency of around 38 MHz with a bandwidth of 83%. Moreover in [3], also using a focused P(VDF-TrFE) transducer and a film thickness of 10 µm, but a conductive epoxy backing, the authors obtained center frequencies around 51 MHz with a bandwidth of 120%. It is interesting to notice that all these reports on focused transducers yield smaller center frequencies than the peak frequencies listed in Table 3, while the bandwidths are either comparable or higher. We believe that these differences are mainly due to variations in physical conditions (e.g., backing material, transducer loading, focusing, and aperture size).

## 8. Conclusions

In all electronic sensor devices, a high conductivity is desired in order to minimize resistive losses that are known to reduce sensor/transducer sensitivity. However, as the current investigation has shown, there will always be a trade-off between increasing the electrode thickness to provide sufficient conductivity, and avoiding unwanted acoustical effects imposed by the electrode thickness. In contrast to previous studies on conductive polymers [8], our test transducers using metal based electrodes with thicknesses typical for printed devices, have identified the electrode mass loading as the most important limiting factor for high frequency performance. Within the range of tested electrode thicknesses (all providing sufficient conductivity), ink-jet printers (or other printing devices) that can produce electrode thicknesses toward the lower thickness range (0.216 μm), will be preferred. Screen printed electrodes, one the other hand, with reported dried thicknesses for nano-based inks from 2 μm and higher [13], might lead to a substantial reduction in the acoustic performance towards the highest frequencies.

To summarize, we demonstrate the feasibility of using an Ag nano-particle ink as electrode material in HF copolymer ultrasonic transducers implemented on a polymer substrate. A number of transducer prototypes were investigated both experimentally and by analytical/numerical methods. The proposed analytic model is particularly useful for optimizing the transducer properties (*i.e.*, sensitivity and bandwidth) for any frequency range and structures where the 1D wave model can be used as an approximation. A relatively good agreement was also established between the suggested constant phase model for the dielectric response and the experimental data. The proposed dielectric model can be used in electrical matching design [28] of the transducer application requiring high sensitivity. The copolymer transducers with Ag nano-particle ink-based electrode were in a pulser-receiver configuration able to send and detect a very broad-banded high-frequency pulse (center frequency >60 MHz and 6 dB bandwidth around 30 MHz or 50%), but with a peak-frequency and magnitude strongly related to the EUT thickness.

## References

[1] Lang S.B., Muensit S. (2006). Review of some lesser-known applications of piezoelectric and pyroelectric polymers. Appl. Phys. A.

[2] Chung C.-H., Lee Y.-C. (2010). Fabrication of poly(vinylidene fluoride-trifluoroethylene) ultrasound focusing transducers and measurements of elastic constants of thin plates. NDT&E Int..

[3] Foster F.S., Harasiewicz K.A., Sherar M.D. (2000). A history of medical and biological imaging with polyvinylidene fluoride (PVDF) transducers. IEEE Trans. Ultrason. Ferr..

[4] Zirkl M., Sawatdee A., Helbig U., Krause M., Scheipl G., Kraker E., Ersman P.A., Nilsson D., Platt D., Bodö P. (2011). An all-printed ferroelectric active matrix sensor network based on only five functional materials forming a touchless control interface. Adv. Mater..

[5] Fuller S.B., Wilhelm E.J., Jacobson J.M. (2002). Ink-jet printed nanoparticle microelectromechanical systems. J. Microelectromech. Syst..

[6] Kim D., Jeong S., Lee S., Park B.K., Moon J. (2007). Organic thin film transistor using silver electrodes by the ink-jet printing technology. Thin Solid Films.

[7] Wagle S., Decharat A., Bodö P., Melandsø F. (2013). Ultrasonic properties of all-printed piezoelectric polymer transducers. Appl. Phys. Lett..

[8] Decharat A., Wagle S., Melandsø F. (2014). Effect of polymer electrode thickness on the acoustical properties of all-screen printed piezoelectric pvdf copolymer transducers. Jpn. J. Appl. Phys..

[9] Sherar M.D., Foster F.S. (1989). The design and fabrication of high frequency poly(vinylidene fluoride) transducers. Ultrason. Imaging.

[10] Kosmala A., Wright R., Zhang Q., Kirby P. (2011). Synthesis of silver nano particles and fabrication of aqueous ag inks for inkjet printing. Mater Chem. Phys..

[11] Okada I., Shimoda K., Miyazaki K. (2006). Development of fine circuit pattern formation process using nano-metal ink. SEI Tech. Rev..

[12] Kim D., Jeong S., Moon J., Kang K. (2006). Ink-jet printing of silver conductive tracks on flexible substrates. Mol. Cryst. Liq. Cryst..

[13] Yin W., Lee D.-H., Choi J., Park C., Cho S. (2008). Screen printing of silver nanoparticle suspension for metal interconnects. Korean J. Chem. Eng..

[14] Joo S., Baldwin D.F. (2010). Adhesion mechanisms of nanoparticle silver to substrate materials: Identification. Nanotechnology.

[15] Greer J.R., Street R.A. (2007). Thermal cure effects on electrical performance of nanoparticle silver inks. Acta Mater..

[16] Park J.-W., Baek S.-G. (2006). Thermal behavior of direct-printed lines of silver nanoparticles. Scripta Mater..

[17] Mason W.P. (1948). Electromechanical Transducers and Wave Filters.

[18] Krimholtz R., Leedom D.A., Matthaei G.L. (1970). New equivalent circuits for elementary piezoelectric transducers. Electron. Lett..

[19] Royer D., Dieulesaint E. (2000). Elastic Waves in Solids i: Free and Guided Propagation.

[20] Royer D., Dieulesaint E. (2000). Elastic Waves in Solids ii: Generation, Acousto-Optic Interaction, Applications.

[21] Wilcox P.D., Monkhouse R.S.C., Cawley P., Lowe M.J.S., Auld B.A. (1998). Development of a computer model for an ultrasonic polymer film transducer system. NDT&E Int..

[22] Brown L.F. (2000). Design considerations for piezoelectric polymer ultrasound transducers. IEEE Trans. Ultrason. Ferr..

[23] Ohigashi H., Itoh T., Kimura K., Nakanishi T., Suzuki M. (1988). Analysis of frequency response characteristics of polymer ultrasonic transducers. J. Appl. Phys..

[24] Westerlund S., Ekstam L. (1994). Capacitor theory. IEEE Trans. Dielect. Electr. Insul..

[25] Ranucci E., Sandgren Å., Andronova N., Albertsson A.-C. (2001). Improved polyimide/metal adhesion by chemical modification approaches. J. Appl. Polym. Sci..

[26] Brown L.F., Carlson R.L., Sempsort J.M. Spin-Cast P(VDF-TrFE) films for high performance medical ultrasound transducers. Proceeding of the IEEE-International Ultrasonic Symposium.

[27] Wagle S., Decharat A., Wenger M., Melandsø F. PVDF copolymer Transducers used to evaluate micro particle suspensions. Proceeding of the IEEE-International Ultrasonic Symposium.

[28] Haiying H., Paramo D. (2011). Broadband electrical impedance matching for piezoelectric ultrasound transducers. IEEE Trans. Ultrason. Ferr..

